# Hospital Meetings

**Published:** 1905-06-10

**Authors:** 


### HOSPITAL MEETINGS.

### HOSPITAL FOR WOMEN.

THE QUESTION OF REMOVAL.

THE annual meeting of the Governors of the Hospital for Women was held on June 1st. The Earl of Shaftesbury, president of the hospital, took the chair.

In moving the adoption of the report he said that the last year having been a particularly fruitful one in legacies for the hospital they had not only been able to pay £500 off the mortgage debt but were left with a balance in hand of £979. But legacies were a precarious source of income, and the general subscription list should be increased. Warm thanks were due to the ladies' committee for their untiring energy and devotion; from the proceeds of their Ball at the Grafton Galleries and otherwise, they had been able to give a contribution of £680 14s. to the hospital. The speaker paid a sympathetic tribute to the memory of Lady Flora Knox, who for over 40 years had been one of the hospital's best friends.

With regard to the recommendation of King Edward's Hospital Fund that the Women's Hospital should be rebuilt on another site, the committee had given the matter their closest consideration and were unanimously agreed that the hospital must be rebuilt but should continue to occupy its present site. Some undesirable premises in the vicinity of the hospital should be bought by the committee and got rid of as opportunity offered.

Mr. F. A. Bevan seconded the motion and spoke of the absolute necessity for rebuilding the hospital and making it up-to-date, otherwise they could not expect the support of either King Edward's Hospital Fund, the Hospital Sunday Fund, or the Hospital Saturday Fund, from all of which they had now grants. It would be an arduous task to raise the £40,000, which was the estimated cost of the rebuilding, but he was sure that great assistance would come from the ladies, who were always so successful in collecting money. It was indispensable that the hospital should remain on its present site, where it was so immensely popular and widely known. From his own experience and connection with other hospitals he thought there was scarcely one where so many applications for treatment were received, which showed how great was the need for the hospital's work amongst poor women. It would be a good thing if millionaires instead of spending fortunes on "blue jars" at Christie's would give a little assistance to this good cause. The presence of the Earl of Shaftesbury, whose grandfather showed also such a kindly interest in hospitals, amongst them that day was a very great pleasure to them all. (Hear, hear.) The report was adopted. A motion that "viewing the rebuilding of the hospital as a necessity to be forcibly recognised it is incumbent on all the governors and supporters of the hospital to unite in an effort to raise sufficient funds to commence the rebuilding within the next twelve months," was moved by Mr. V. A. Williamson, who said that after the report of King Edward's Fund there was no doubt but that the rebuilding must go forward—the only question was to change or not to change the site? Many persons whose opinions were of moment were in favour of transportation to an airy suburb—the claims of about twenty possible sites were therefore investigated—and none of them were found to possess equal advantages with the one at present occupied by the hospital. It was therefore conclusive that they must rebuild where they were, and their architect, Mr. Adams, had now submitted the plans for the rebuilding. Mr. Harvie seconded the motion. As responsibility would have to be taken for the whole £40,000, he hoped that £20,000 at least would be given to the committee to start with. The resolution was carried.

A vote of thanks to the Earl of Shaftesbury for presiding having been unanimously carried, the meeting ended.

### METROPOLITAN HOSPITAL.

THE Governors held their annual meeting in the Boardroom of the Metropolitan Hospital on June 5th, and were presented with the sixty-ninth annual report of the Committee of Management. Until the arrival of Lord Howard de Walden the chair was temporarily taken by Mr. Charles J. Thomas (chairman of the Committee of Management), who moved the adoption of the report. He said that in spite of a deficit in the ordinary income of £4,106 over that of 1903, yet the committee were glad to say that 1904 had been a successful year. They had an increase of £3,258 2s. 3d. from legacies, and the total sum realised under this heading, £4,508 2s. 3d., was almost the largest they had received since the opening of the new buildings in 1887. One of the most important events in the hospital's life during the last year had been the installation of electric light throughout, and the construction of an electric lift. For both these benefits the committee were indebted to the kindness of the executors of the late Mr. Arthur Ocran Crooke, who also, out of the discretionary powers left them by the will, have endowed a bed in the hospital in memory of the testator. Sincere thanks were also due to Mr. A. Lister Harrison for the erection of 25 inter-communicating telephones through the hospital. The committee were sorry to lose the valuable services of several of their nurses who had, however, mostly resigned their positions on account of good promotion. Two or three were unfortunately obliged to leave through ill-health, and one was going to be married. Decided benefit has accrued from the almoner's successful carrying out of his work, and the committee report that the nurse appointed upon the advice of the Medical Committee to visit the hospital's patients in their own homes and give them some instruction on (1) the care of children; (2) food and feeding; (3) sanitation and general hygiene, has entered upon her work. As £14,000 has to be raised for working expenses every year the committee sincerely hope that the public will keep the urgent needs of the hospital in view.

The motion was seconded by Mr. J. R. Pike—who remarked that the report reflected credit on all concerned—and was unanimously adopted.

### FRIEDENHEIM HOSPITAL.

THE annual meeting of the subscribers to the Friedenheim Hospital took place on the 3rd inst. in the Hampstead Conservatoire. The chair was taken by Mr. John Langton, F.R.C.S. (Chairman of the Council), who said that, in spite of many difficulties and trials and of dark days of financial trouble, those who were carrying on the work of the hospital always felt that God's blessing was resting upon them and upon their efforts. Financial matters were now improving, for they were glad to say that the building itself was at last free from debt; and though at the end of 1904 the deficit was £614, yet this sum showed an improvement of £207 on the previous year. But to keep the building free from debt increased annual subscriptions were needed. He said that though generally the dying persons admitted had but a short time to stay, they nevertheless found preparation in the hospital for calm and peaceful deaths, and he earnestly commended the Friedenheim Hospital to the liberality and prayers of its friends.

The Bishop of London, in proposing the adoption of the report and a vote of thanks to all who had assisted in the work of the institution during the past year, expressed the great pleasure it gave him to support the Christian work which the hospital was doing. Nine years of his own clerical life had been spent in the slums of London, and there he had seen the sordidness, the noise, the wretchedness amidst which the poor must die, while here Friedenheim transposed them to sunshine and calm and loving care, and thus during their last days on earth gave them a foretaste of the peace of Heaven.

The Duchess of Montrose seconded the resolution and said she knew few institutions so worthy of support as this Home of Peace. All possible means were taken to soothe and beautify the patients' remaining days—and certainly the work of those who carried out this mission was beyond all praise. Illness united with poverty was doubly a trial, and the sick poor, for their families' sakes, were generally most anxious to be removed from their homes.

The resolution was unanimously adopted.

A vote of thanks to the Bishop of London and to the Duchess of Montrose was very cordially moved by Prebendary Webb-Peploe, who added that the presence of her Grace that day proved that the charges brought against Society of frivolity and uselessness had very notable exceptions in their application.

Dr. A. T. Schofield, in seconding the motion, said that a most important portion of the hospital's work which had not hitherto been touched upon, was the good work they did in preventing the spread of pulmonary disease by the sequestration within their walls of dying consumptives. Patients sometimes became better; he mentioned one notable case which, certified to be hopeless, nevertheless perfectly recovered.

The resolution was unanimously carried.

### NORTH-EASTERN HOSPITAL FOR CHILDREN.

The annual meeting of the governors of the North-Eastern Hospital for Children was held in the Board-room of the hospital on May 31st.

In the unavoidable absence of Lord William Cecil, Mr. Walter Johnson, J.P., took the chair. He referred to the grave financial crisis in the hospital's affairs which had confronted the governors at their last court and to the much happier auspices under which the present meeting was being held; great care was being taken, and a special committee had been appointed to see, that the administration of the hospital should be as economical as was compatible with perfect efficiency. The erection of the much-needed nurses' home and laundry were now to be proceeded with; the buildings would cost about £10,000, of which they already had £7,000 in hand. The nurses' home was to be first dealt with, but on completion of both the saving in the hospital's expenditure would be about £800 per annum. Another benefit was the great comfort and convenience for the nurses in at last having their quarters close to their hospital.

The Rev. J. E. Watts-Ditchfield, proposing the adoption of the report, said that from his own experience as vicar of a very poor district he could prove the intense need and necessity for the hospital's work. The speaker pictured the sufferings of little children sick and crying amidst surroundings of the direst poverty, and added, that as the very poorest in the district had done their best to help their hospital in its time of stress, they had certainly some claim upon the practical sympathy of the rich.

The Rev. J. Hillman seconded the motion, which was unanimously carried.

The Chairman, introducing Mr. Charles Pont as the mover of a vote of thanks to H.R.H. Princess Henry of Battenberg, President, Lady Ampthill, Vice-President, the Hon. Constance Russell, Hon. Sec., and other members of the Ladies' Association for assistance given in arranging, and to the Lord Mayor for presiding at, the Mansion House luncheon on May 22nd, spoke in the highest terms of the great time and devotion which Mr. Pont had dedicated to the service of the hospital.

A motion for the election of the officers and committee for the ensuing year having been moved by Mr. Alfred Clarke, seconded by Mr. Butterfield, and adopted, Mr. Joseph Miller, in moving a vote of thanks to the staff, gave some striking instances of their individual devotion during the hospital's financial crisis. He gave special praise to the splendid work of the secretary, and returned thanks for what had been said of himself.

Mr. Martin Deed, having seconded, the Secretary, Mr. T. Glenton-Kerr, responded, and the motion was unanimously carried.

A vote of thanks to the Chairman was also unanimously accorded, and the meeting concluded.

